# Genetic basis of brain size evolution in cetaceans: insights from adaptive evolution of seven primary microcephaly (*MCPH*) genes

**DOI:** 10.1186/s12862-017-1051-7

**Published:** 2017-08-29

**Authors:** Shixia Xu, Xiaohui Sun, Xu Niu, Zepeng Zhang, Ran Tian, Wenhua Ren, Kaiya Zhou, Guang Yang

**Affiliations:** Jiangsu Key Laboratory for Biodiversity and Biotechnology, College of Life Sciences, Nanjing Normal University, 1 Wenyuan Road, Nanjing, 210023 China

**Keywords:** Cetacea, *MCPHs*, Positive selection, Brain size evolution, EQ, Group size

## Abstract

**Background:**

Cetacean brain size expansion is an enigmatic event in mammalian evolution, yet its genetic basis remains poorly explored. Here, all exons of the seven primary microcephaly (*MCPH*) genes that play key roles in size regulation during brain development were investigated in representative cetacean lineages.

**Results:**

Sequences of *MCPH*2–7 genes were intact in cetaceans but frameshift mutations and stop codons was identified in *MCPH1*. Extensive positive selection was identified in four of six intact MCPH genes: *WDR62*, *CDK5RAP2*, *CEP152*, and *ASPM*. Specially, positive selection at *CDK5RAP2* and *ASPM* were examined along lineages of odontocetes with increased encephalization quotients (EQ) and mysticetes with reduced EQ but at *WDR62* only found along odontocete lineages. Interestingly, a positive association between evolutionary rate (ω) and EQ was identified for *CDK5RAP2* and *ASPM*. Furthermore, we tested the binding affinities between Calmodulin (CaM) and ASPM IQ motif in cetaceans because only CaM combined with IQ, can ASPM perform the function in determining brain size. Preliminary function assay showed binding affinities between CaM and IQ motif of the odontocetes with increased EQ was stronger than for the mysticetes with decreased EQ. In addition, evolution rate of *ASPM* and *CDK5RAP2* were significantly related to mean group size (as one measure of social complexity).

**Conclusions:**

Our study investigated the genetic basis of cetacean brain size evolution. Significant positive selection was examined along lineages with both increased and decreased EQ at *CDK5RAP2* and *ASPM*, which is well matched with cetacean complex brain size evolution. Evolutionary rate of *CDK5RAP2* and *ASPM* were significantly related to EQ, suggesting that these two genes may have contributed to EQ expansion in cetaceans. This suggestion was further indicated by our preliminary function test that *ASPM* might be mainly linked to evolutionary increases in EQ. Most strikingly, our results suggested that cetaceans evolved large brains to manage complex social systems, consisting with the ‘social brain hypothesis’, as evolutionary rate of *ASPM* and *CDK5RAP2* were significantly related to mean group size.

**Electronic supplementary material:**

The online version of this article (doi:10.1186/s12862-017-1051-7) contains supplementary material, which is available to authorized users.

## Background

Cetaceans are a group of secondary-adapted marine mammals, the common ancestor of which diverged from terrestrial artiodactyls approximately 53–56 million years ago (Ma) [1]. Cetaceans comprise of one extinct (Archaeoceti) and two extant (Mysticeti and Odontoceti) suborders. Until 40 Ma, archaeocetes were completely aquatic [2]. Extant cetaceans evolved from archaeocetes at about 34 Ma, and distributed nearly all the world’s oceans, as well as some freshwater lakes and rivers [1]. During the transition from terrestrial to fully aquatic environments, significant changes affecting sensory systems, locomotion, breathing and feeding took place [3], of which, the large brains of modern cetaceans remains the most enigmatic [4].

Fossil and anatomical evidence show that the brain size (or encephalization) of archaeocetes was similar to their ancestor [5]. The brain mass of mysticetes increased, but their body mass increased at a much rapid rate leading to a decrease in relative brain size (as measured by encephalization quotient, i.e. EQ, which accounts for body size) with a mean EQ of 0.21 [6]. Reduced EQ was suggested to be related to the massive biomechanical forces needed to open their mouths when feeding [7]. In contrast, the relative brain size of the odontocetes (mean EQ = 3.10) is higher than that of their ancestors (mean EQ = 2.43) [6]. For example, some species of delphinids have EQs (4–5) significantly larger than nonhuman primates (EQ = 3.3) and are second only to humans (EQ = 7) [4]. What kinds of selective pressures could have led to this rapid increase in brain size among odontocetes? Previous ecological studies have shown that odontocetes have high degrees of encephalization primarily as an adaptation for living in complicated social groups (cooperative actions and fission–fusion societies), as asserted by the ‘social brain hypothesis’ [6, 8–12]. This hypothesis is supported by the positive correlation between EQ and one measure of social complexity, group size, in many dolphin species [13]. However, the brain size evolution in cetaceans remains poorly tested at the molecular level.

There has been much interest in exploring the genetic basis of adaptive phenotypes using candidate genes or gene families. Primary microcephaly (*MCPH*) genes are thought to play key roles in size regulation during brain development in mammals due to the fact that *MCPH* gene mutations can cause severe defects in the development of cerebral in humans [14, 15]. So far, seven autosomal recessive loci (*MCPH1*–*7*) have been identified in humans: *MCPH1*, *WDR62*, *CDK5RAP2*, *CEP152*, *ASPM*, *CENPJ* and *STIL* [15]. Specially, there is a 1:1 orthologous relationship of these genes in dolphins and whales. Increasingly, evidence of positive selection was detected at *MCPH* gene, special for *ASPM*, *CDK5RAP2* and *MCPH1* genes, across primate lineages with massive brain size [16, 17]. A recent study suggested that *ASPM* was linked to both evolutionary increases and decreases in brain size in anthropoids with positive selection acting on both lineages [18]. Investigating the genetic basis of brain size evolution in cetaceans was only recently commenced with examination of some exons of the *MCPH1* and *ASPM* genes in cetaceans [19, 20]. Evidence of positive selection was determined on exons 3 and 18 of the *ASPM* gene in odontocetes, especially for species in the superfamily Delphinoidea, which was well matched with the two major events of relative brain size enlargement in cetaceans [20]. However, no significant association was identified between the evolutionary rate of the two *ASPM* exons and brain size phenotypes in cetaceans [21]. For *MCPH1* gene, no compelling evidence of positive selection and association was examined between *MCPH1* evolution and brain evolution in cetaceans [19]. Different results from these two *MCPH* genes suggest a complex mechanism of brain size evolution in cetaceans. Here, the evolution of seven *MCPH* genes was investigated in representative species of major cetacean lineages. First, we tested whether different selection patterns acted on the seven *MCPH* genes in cetacean lineages and whether positive selection was limited to lineages with high EQs. Second, we explored the putative association between the evolutionary rate of *MCPH* genes and some morphological variables of cetacean brains. Third, the correlation between *MCPH* evolution and group size was examined to test support for the ‘social brain hypothesis’ at the molecular level.

## Methods

### *MCPH* genes and primary treatments

A total of 16 cetacean species (three mysticetes and 13 odontocetes) was used in our study (see Additional file 1: Table S1). Of them, samples of 13 species (two mysticetes and 11 odontocetes) were collected from dead individuals in the wild and no ethics statement was required in such occasions. We first downloaded the full-length coding sequence (CDS) of seven *MCPH* genes from the database of Orthologous Mammalian Markers (http://www.orthomam.univ-montp2.fr/orthomam/html/) and designed the primers to amplify each exon of seven *MCPH* genes. We then sequenced these exons in 13 samples and merged into the predicted full-length CDS. Species information, genomic DNA extraction, primer design, PCR amplification and sequencing were conducted as described in Xu et al. [20]. The *MCPH* orthologous gene sequences from the other three cetacean species (i.e. bowhead whale *Balaena mysticetus*, killer whale *Orcinus orca*, and sperm whale *Physeter macrocephalus*) and two terrestrial relatives (Hippopotamus *Hippopotamus amphibius* and cow *Bos taurus*) were available from their published genomes (see Additional file 1: Table S1). We used two alignment methods, i.e. CLUSTAL and MUSCLE, as implemented in MEGA 6.0 (Tamura et al. [22]) to align the nucleotide sequences of each *MCPH* gene and verified by visual inspection.

### Molecular evolution analysis

The nonsynonymous (*d*_N_) / synonymous substitution (*d*_S_) rate (ω = *d*_N_ / *d*_S_) is a measure of selective pressure, with values of ω >1, = 1, and <1 indicating positive selection, neutral selection, and purifying selection, respectively. The ω ratios were estimated using the codon-based maximum likelihood (ML) models implemented in the CODEML program in PAML 4.4 [23]. A well-accepted phylogeny of Cetacea [24] was used as input tree in our analysis for each gene. The phylogenetic trees were also reconstructed using the maximum-likelihood (ML) and Bayesian inference (BI, See the Additional file 2). The gene trees were similar to the well-accepted phylogeny with only some minor differences within Delphinidae (see the Additional file 2: Figure S1). According to Yang et al. [25] suggestions, the minor differences in the phylogeny do not make any significant difference in identification of positively selected sites. Hence, selection detection using the gene trees produced results similar to those obtained using the well-accepted phylogeny of Cetartiodactyla (see the Additional file 1: Table S2), only the latter result was reported here.

To examine the probabilities of sites under positive selection in the six intact genes, we first used two pair of site models: M7 (beta) versus M8 (beta & ω_2_ > 1) [26], and M8a (beta & ω_2_ = 1) versus M8 [27] implemented in the CODEML program of PAML 4.7. The nested models were compared using a likelihood ratio test (LRT) with a χ^2^ distribution. Positively selected sites in the M8 were identified using a Bayes Empirical Bayes (BEB) analysis [28] with posterior probabilities ≥0.80. Positive selected sites were further detected by fixed effects likelihood (FEL) performed in HYPHY [29] (via the www.datamonkey.org web server), with the default settings with significance levels of 0.2. We then performed selective pressure detection using TreeSAAP v.3.2 [30], which detected selection based on 31 physicochemical amino acid properties. All magnitude category 6–8 changes with *P* values ≤0.05 were used as an index for the degree of radical amino acid substitution and positive selection.

To evaluate whether positive selection was restricted to specific cetacean lineages, we used branch models (including free-ratio model and two-ratio model [31, 32]) and branch-site model implemented in CODEML [23]. The free-ratio model (M1) that assumes an independent ω ratio for each branch was compared with the null one-ratio model (M0) with the same ω for all branches [31]. Two-ratio model and branch-site model require the foreground branches (lineages tested to be under positive selection) and background branches (rest of the lineages) to be defined a priori. Each cetacean lineage across the Cetartiodactyla phylogeny was used as the foreground branch, respectively, whereas the remaining branches were treated as background branches for each gene. We compared the two-ratio model where ω was allowed to differ in the background and a foreground branch with null M0 model [31, 32]. By contrast, the branch-site model appeared to be conservative but far more powerful than the branch-based model. The modified branch-site model A with ω varying among sites and among lineages [28, 33] was tested against the recommended null hypothesis of no selection in any of the foreground or background branches. According to Zhang et al. [33], sites identified by this method can still be evidence of positive selection even if the BEB cannot be reliably inferred because the tested positive selection at any single site may not be strong enough for the BEB probability to reach high levels if positive selection has affected only one lineage or a very few lineages on the tree. A false discovery rate (FDR) correction for multiple tests was applied to the LRT *P* values for branch-site model analysis [34].

### Association analysis between gene evolution and phenotypes

To explore potential relationships between the evolutionary rate (ω) of *MCPH* genes and brain size phenotypes we used the method of Montgomery et al. [16] whereby the root-to-tip ω is regarded as more suitable for regression against phenotypic data from extant species because the root-to-tip ω is more inclusive of the evolutionary history of a locus [35]. The following phenotypic traits including absolute brain mass and absolute body mass from 11 cetacean species were derived from published data [5, 32–39] (see Additional file 1: Table S3). EQ values for each species were derived from the eq. EQ = brain weight/0.12 (body weight) ^0.67^ from Jerison [40]. We then used phylogenetic generalized least squares (PGLS) regression, performed in R 3.1.2 using the packages Caper [41], to analyze the relationship between log-transformed (root-to-tip ω) and each log-transformed morphological variables. The detailed analytical procedures were provided in the supplementary material online (see Additional file 2).

Social complexity may be as a major force for brain evolution in cetaceans [12]. When group size is used as a measure of social complexity, one should expect to see strong relationships between group size and the evolutionary rates of *MCPH* genes in cetacean species. Thus, PGLS regression analyses were also used to test whether there was association between mean group size and gene evolution of such *MCPH* genes subject to positive selection.

### Three-dimensional (3D) structure prediction

To provide further insights into the functional significance of these positively selected sites, they were mapped onto the three-dimensional (3D) structures of *MCPH* genes using PYMOL (http://pymol.sourceforge.net/). We first predicted the 3D structures of *MCPH* genes following homology modeling using the SWISS-MODEL (http://swissmodel.expasy.org). However, no significant amino acid sequence similarity with known proteins or no consistent results were detected using BLAST tools. Thus, an ab-initio 3D model of each *MCPH* gene was constructed by I-TASSER [42, 43], a state-of-the-art hierarchical protein structure modeling approach based on secondary-structure enhanced profile-profile threading alignment [42].

### Functional assays of the *ASPM* gene

Bioinformatics analyses provided a series of support for the positive selection on *MCPH* genes, especially *CDK5RAP2* and *ASPM*, in the brain size enlargement or decrease of cetaceans. However, considering that these genes were first identified in humans, it would be better to provide some additional functional evidences which could not only suggest these genes do have function in cetaceans, but further support their important roles in brain size evolution that were revealed through bioinformatics analyses. In the present study, we chose *ASPM* gene as a representative to conduct functional experiments and expected to present partial and preliminary evidences.

The *ASPM* gene is a major determinant of cerebral cortical size [44]. Four distinguished regions were identified in the predicated *ASPM* gene in human, comprising a putative microtubule-binding domain, a calponin-homology domain, an IQ repeat domain containing 81 IQ motifs (CaM-binding motifs), and a C-terminal region [44]. Of these, CaM-binding IQ motifs were suggested to play an essential role in determining brain size [45]. Considering that the CDS of the cetacean *ASPM* gene was more than 10,300 bp in CDS with 28 exons of the bottlenose dolphin *T. truncatu*, the expression vector cannot carry such a large DNA fragment. Thus, only the one IQ motif (the 23rd IQ when the bottlenose dolphin *Tursiops truncates* as reference) including one positively selected site, i.e. 1684) and its adjacent 16 amino acid motif were cloned into the expression vector. Six species of odontocetes with increased EQ and three species of mysticetes with reduced EQ were chosen as representative samples in this study.

We then used GST pull-down assay to evaluate if CaM interact with IQ motif in cetacean lineages with brain enlargement or decrease. Binding affinities between CaM and IQ motif were further quantitatively determined by biolayer interferometry (BLI) using the ForteBio Octet Red system. Binding affinities were calculated using ForteBio Data Acquisition 6.3 software (ForteBio). Equilibrium dissociation constants (*K*d) were calculated as the ratio of dissociation and association rate constants (*K*off /*K*on). All detailed experiment procedures were listed in the Additional file 2.

## Results

Almost all exons of the seven *MCPH* genes were successfully amplified in 13 representative species of cetaceans. Newly obtained sequences for each *MCPH* gene (GenBank accession nos. KY011963- KY012055) covered at least 74.16% of the full CDS: 86.31% for *MCPH1*, 85.68% for *WDR62*, 74.78% for *CDK5RAP2*, 88.91% for *CEP152*, 90.34% for *ASPM*, 74.16% for *CENPJ* and 87.86% for *STIL*. Evidence of intact gene was identified in cetaceans at six genes (*MCPH*2–7) because non-frameshift insertions/deletions and premature stop codons were observed in sequences of these genes. However, *MCPH1* has been pseudogenized in some cetacean lineages because premature stop codons were identified in three species (i.e. beluga *Delphinapterus leucas*, Risso’s dolphin *Grampus griseus* and killer whale *O. orca*) and frameshift insertions/deletions were examined in five cetacean species (including Blainville’s beaked whale *Mesoplodon densirostris*, dwarf sperm whale *Kogia sima*, Chinese white dolphin *Sousa chinensis*, *G. griseus*, and *P. macrocephalus*). We exclude the possibility that frameshift insertions/deletions and stop codons are the result of sequencing error because we have reamplified these pseudogenized fragment of *MCPH1* gene in different samples or using different primers and obtained the same result. Thus, the six intact genes of *MCPH* were used for our further analyses.

### Selection on MCPH genes

We found M8 that incorporated selection fit the data better than the neutral model, M8a, at the four *MCPH* genes (*WDR62*, *CDK5RAP2*, *CEP152*, and *ASPM*; *P* < 0.001), suggesting that these genes were subjected to positive selection in cetaceans. However, for *CENPJ* and *STIL*, associated the LRT showed no significant difference between the models M8 and M8a (*CENPJ*: *P* = 0.485; *STIL*: *P* = 0.195), implying no positive selection. Using M8, the most stringent model carried out in PAML, a small proportion of codons (1.82–7.57%) were estimated to be under selection with average ω values of 4.438–10.203 at the four positively selected genes in cetaceans (Table 1). Seven, 39, 10, and 20 positively selected sites were identified by the BEB approach as having posterior probabilities ≥0.80 at *WDR62*, *CDK5RAP2*, *CEP152*, and *ASPM*, respectively (Table 1). When we used a significance threshold of 0.95 for posterior probabilities, the number of positive selected amino acids decreased to three, nine, six, and four at the four genes, respectively. FEL, performed in HYPHY [24], was also used to test for selection in the six intact *MCPH* genes. HYPHY can improve the estimation of the ω value by incorporating variation in *d*_S_ whereas *d*_S_ is fixed across sequences for all the PAML-based analysis [23, 24]. FEL analysis showed that significant signs of positive selection were detected at the six *MCPH* genes with many more positively selected sites than that identified by M8 (Table 1). Combining the two different maximum likelihood (ML) methods, a total of 36 positively selected sites (six at *WDR62*, 16 at *CDK5RAP2*, six at *CEP152*, and eight at *ASPM*) were picked out (Table 1). Sites identified to be under positive selection by two ML methods were regarded as robust candidates for sites under selection. Therefore, 36 robust sites under positive selection were used in our next analyses. We further employed a complementary protein-level approach implemented in TreeSAAP [30] to evaluate destabilizing radical changes at each robust site. The result showed that 32 of 36 sites (88.89%) have radical changes in at least one property whereas 16 sites (44.44%) had at least three changes in properties at the four *MCPH* genes (see Additional file 1: Table S4). When an empirical threshold of *P* ≤ 0.05 was applied we found 13 sites (36.11%) under strong positive selection at the protein-level (see Additional file 1: Table S4).

**Table 1:** Positively selected sites detected using two maximum likelihood (ML) methods across cetacean phylogeny

Gene	Test of Selection						Sites under Selection Identified by ML Methods			
	-Ln (M8a)	-Ln (M7)	-Ln (M8)	−2△L (M7 vs.m8)	−2△L (M8a vs.m8)	ω value	PAML M8^a^	FEL^b^	no. of Sites^c^	% of Sites
*WDR62* (1298aa)	7703.049	7703.257	7690.821	24.872*	24.456*	10.203	**262**, **458**, **479**, **972**, **1008**, **1133**, 1244	207, **262**, **458**, **479**, 648, 705, 763, **972**, **1008**, 1076, 1092, 1107, **1133**, 1158	6	0.46%
*CDK5RAP2* (1414aa)	10,316.782	10,317.746	10,285.732	64.082*	62.101*	4.438	**23**, **108**, **182**, **201**, **283**, 382, 394, **426**, **445**, 518, 523, **531**, 558, 615, 678, 686, 700, 717, **720**, 724, 726, 727, 740, **760**, 763, 786, **787**, **832**, **849**, **913**, 982, 1012, 1020, 1030, **1166**, 1167, **1284**, 1370, 1396	6, 10, **23**, 30, **108**, 174, **182**, **201**, 204, **283**, **426**, 427, **445**, **531**, **720**, **760**, **787**, **832**, **849**, 887, **913**, **1166**, 1280, **1284**, 1310, 1313	16	1.13%
*CEP152* (1523aa)	9948.922	9949.080	9939.249	19.662*	19.347 *	7.312	**79**, **305**, 477, **510**, 543, 613, **1147**, **1163**, 1360, **1398**	73, **79**, 239, **305**, 328, 401, **510**, 717, 941, 966, 1088, 1097, **1147**, **1163**, 1281, **1398**	6	0.39%
*ASPM* (3128aa)	20,714.804	20,714.924	20,700.708	28.432*	28.191*	5.236	**69**, **347**, **387**, 595, **692**, 805, 806, **1311**, **1602**, 1684, 1787, **1864**, 2096, 2098, 2350, **2619**, 2693, 2798, 2819, 3111	22, 23, **69**, **347**, **387**, 542, 677, **692**, 1178, **1311**, **1602**, 1657, 1660, 1682, 1737, 1741, 1758, **1864**, 2106, 2187, 2205, 2445, 2465, 2507, 2510, 2546, **2619**, 2817, 3098	8	0.26%
*CENPJ* (998aa)	7086.280	7086.473	7086.040	1.700 (NS)	0.488 (NS)	1.593	**__**	112, 366, 370, 451, 473, 488, 499, 513, 544, 567, 645	**__**	**__**
*STIL* (1125aa)	6173.275	6173.287	6172.437	0.866 (NS)	1.677 (NS)	4.921	**__**	471, 575, 991	**__**	**__**

The positively selected sites picked out by two methods are shown in bold

* indicates *P* < 0.05 whereas NS indicates ‘No significant’

^a^Codons were detected by M8 model in PAML using a Bayes Empirical Bayes (BEB) analysis with posterior probabilities ≥0.80

^b^Condons was determined by FEL implemented in HYPHY with significance levels of 0.2

^c^No. of sites indicate positively selected sites identified by both ML methods

**Fig. 1:**
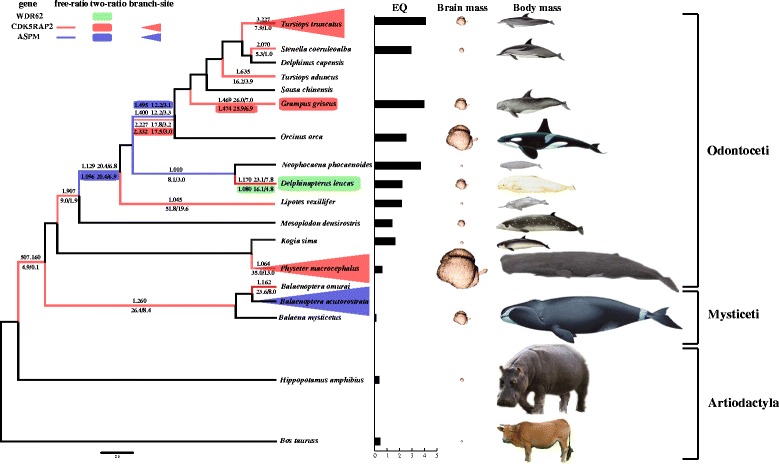
Evidence of positive selection across the phylogeny of cetartiodactyla identified by branch and branch-site models. The three genes identified to be under positive selection are marked with different colors: *WDR62* (green), *CDK5RAP2* (pink), and *ASPM* (blue). Significant positive selection identified by free-ratio, two-ratio and branch-site models are highlighted and indicated by line, rectangle and triangle, respectively. The ω value greater than 1 for individual lineage according to free-ratio and two-ratio are shown

To evaluate whether positive selection is only limited to particular lineages at the six intact *MCPH* genes, we first used branch models (including free-ratio and two-ratio models) that allow the ω ratio to vary among branches across the phylogeny. The LRT tests showed that evidence of positive selection was detected at the *WDR62*, *CDK5RAP2* and *ASPM* genes whereas no selection was detected for the other three genes. Using a free-ratio model, we found that ω was greater than 1 in branches of odontocetes with increased EQ at *ASPM*: last common ancestor (LCA) of delphinids, LCA of the Baiji *Lipotes vexillifer*, and LCA of the Indo-Pacific finless porpoise *Neophocaena phocaenoides* and beluga *D. leucas* (Fig. 1). In contrast, evidence of positive selection was examined at *CDK5RAP2* in lineages both with increased or decreased EQ. That is, a ω greater than 1 was found along lineages leading from the LCA of cetaceans to odontocetes with increased EQ (terminal branch of *P. macrocephalus*, LCA of delphinids, LCA of *M. densirostris*, branch leading to *L. vexillifer*, terminal branch of *D. leucas*, LCA of delphinids, and four terminal branches of delphinids) and the ancestral branch of mysticetes and branch leading to Omura’s whale *B. omurai* with reduced EQ. In the two-ratio model, significant signs of positive selection were restricted to lineages with expanded relative brain size, such as the LCA of delphinids at both *CDK5RAP2* and *ASPM*, LCA of *L. vexillifer* at *ASPM*, the branch leading to *G. griseus* at *CDK5RAP2*, and the branch leading to *D. leucas* at *WDR62* (Fig. 1). Similar results were obtained with the stringent branch-site model, which revealed that two lineages with increased EQ (such as the terminal branch of *T. truncatus* and *P. macrocephalus*) at *CDK5RAP2* and one with reduced EQ (i.e. branch leading to *B. acutorostrata*) at *ASPM* were subject to selection after FDR correction, respectively (Fig. 1). In addition, seven and one positively selected sites were identified at *CDK5RAP2* in branches leading to *T. truncatus* and *P. macrocephalus*, respectively. However, none were found at *ASPM* even using the posterior probabilities ≥0.50 as the cutoff for the positively selected sites.

### Assocition between *MCPH* evolution and morphological variables

**Fig. 2:**
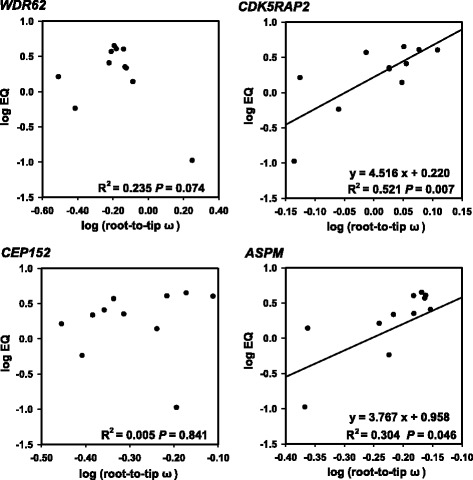
Regression analyses between root-to-tip ω and EQ across cetacean phylogeny

To explore the associations between the evolution of each *MCPH* gene found to be under positive selection (represented by root-to-tip ω) and absolute brain mass, body mass and EQ (see Additional file 1: Table S3), we performed PGLS regressions, as implemented in the R 3.1.2 using the packages Caper [41]. Regression analyses revealed a positive association between log (root-to-tip ω) and log (EQ) at *CDK5RAP2* (*R* ^2^ = 0.521, *P* = 0.007) and *ASPM* (*R* ^2^ = 0.304, *P* = 0.046, Fig. 2; see Additional file 1: Table S5), whereas no such association was found for the other two *MCPH* genes under positive selection, i.e. *WDR62* (*R* ^2^ = 0.235, *P* = 0.074) and *CEP152* (*R* ^2^ = 0.005, *P* = 0.841). In addition, log (root-to-tip ω) was not related to brain mass and body mass for all of the four *MCPH* genes under positive selection (see Additional file 1: Table S5).

### Relationship between ω and mean group size

**Fig. 3:**
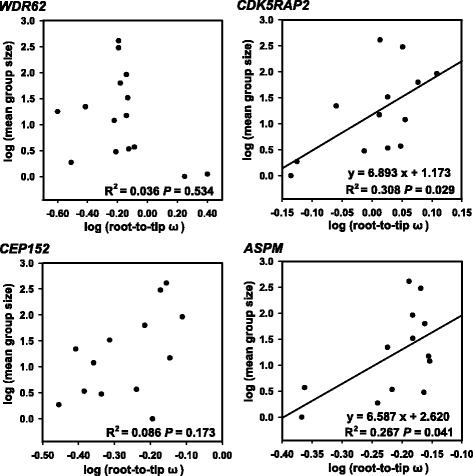
Regression analysis between root-to-tip ω and mean group size in cetaceans

To test whether social complexity drove cetacean brain size expansion we used mean group size as a measure of social complexity. Mean group size from 13 cetacean species used in our study was derived from May-Collado et al. [46] (see Additional file 1: Table S3). Regression analyses showed a significant association between log (root-to-tip ω) and log (mean group size) for *ASPM* (*R* ^2^ = 0.267, *P* = 0.041) and *CDK5RAP2* (*R* ^2^ = 0.308, *P* = 0.029), whereas no significant association was found for *WDR62* (*R* ^2^ = 0.030, *P* = 0.574) and *CEP152* (*R* ^2^ = 0.086, *P* = 0.173) (Fig. 3; see Additional file 1: Table S6).

### Spatial distribution of positively selected sites in 3D structures of MCPH genes

**Fig. 4:**
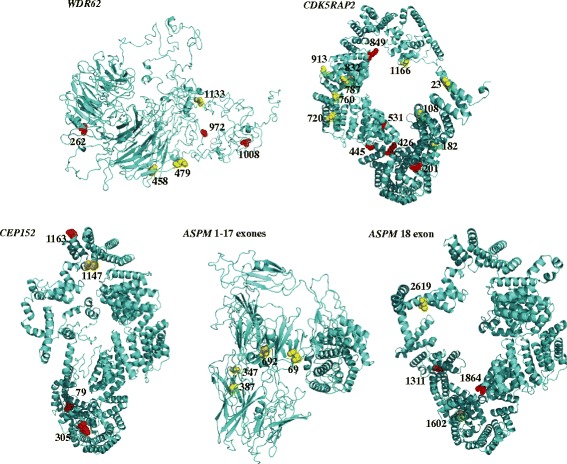
Radical amino acid changes in selected sites mapped on the three-dimensional structure of four *MCPH* genes in cetaceans. Radical changes of positively selected sites identified by two ML methods are colored with yellow ball and red ball whereas those positively selected sites with significant radical changes are marked with red ball

A total of 32 radical amino acid changes subjected to positive selection identified by two ML methods were mapped onto the 3D structures of four *MCPH* genes (six at *WDR62*, 14 at *CDK5RAP2*, four at *CEP152*, and eight at *ASPM*). We found that 16.67%–87.5% of positively selected sites were localized in the functional region in the predicted 3D structure of each *MCPH* gene (Fig. 4). For example, for *ASPM*, up to 87.50% of positively selected sites (7 / 8) were localized in two putative key functional domains: the CaM-binding IQ motifs (1311, 1602, 1864, and 2619) and microtubules domain (69, 347 and 387). For *CDK5RAP2*, eight sites (50%) were scattered over the three predicated functional regions: one (23) in the γTurc binding domain, three (182, 201, and 283) in the structural maintenance of chromosomes (SMC) domain, and another four (720, 760, 787, and 832) in SMC_N domain, that were known to play a key role in the cohesion and condensation of chromosomes during mitosis. Only two (262, 458) or one (305) sites were localized in the functional region of *WDR62* (WD40 repeat domain) and *CEP152* (coiled_coil domain), respectively.

### Functional assay of *ASPM*

**Fig. 5:**
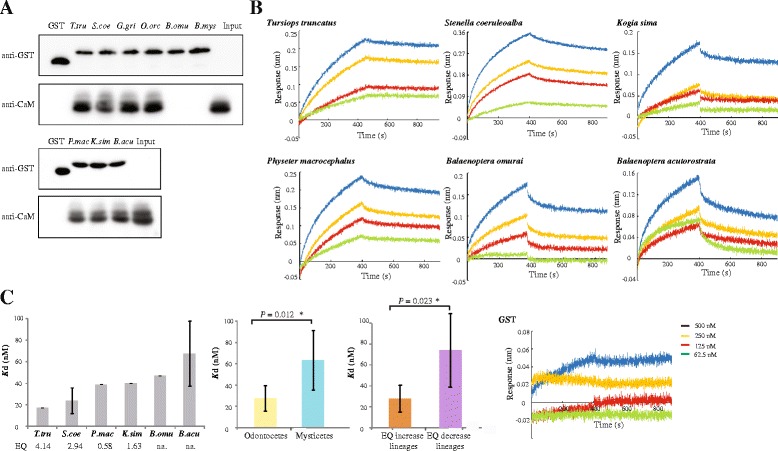
In vitro assay for protein-protein interaction between *ASPM* IQ motif and calmodulin (CaM). **a** GST pull-down assay indicating a possible interaction of the *ASPM* IQ motif and CaM. Latin name of species shorthand initial capital letter for the genus and the first three letters of species listed upper gel-image. **b** Quantitative analysis of interaction of *ASPM* IQ and CaM using BLI analysis. The association and dissociation of increasing concentrations of *ASPM* IQ motif to CaM were shown. **c** Comparison of dissociation constants (*K*d) values derived from BLI analysis. *P* values were calculated by Student’s t-test

Previous studies showed that the IQ motifs, which act as calmodulin-binding domains, were thought to be involved in increased cerebral cortical size in mammalian evolution (reviewed in ref. [15]). Considering that the coding sequence of the cetacean *ASPM* gene was more than 10,300 bp, the expression vector cannot carry such a large DNA fragment. Thus, only the 23rd IQ motif (66 bp) including one positively selected site, i.e. 1684) and its adjacent 16 amino acid motif were cloned into the expression vector. We performed a GST pull-down assay probed by immunoblotting with GST-IQ as bait and His-CaM as prey. The result showed that this IQ motif in the six odontocete species bound to CaM (Fig. 5a). In contrast, the IQ motif of mysticetes did not bind to CaM except for *B. acutorostrata*. Further, four odontocete and two mysticete species were chosen to measure the binding affinities of the GST-IQ protein and CaM using BLI analysis that can quantitatively analyze protein interactions in real-time. BLI analysis revealed that the CaM could effectively bind to GST-IQ fusion protein of both odontocetes and mysticetes, but not the GST control (Fig. 5b). The affinities of the GST-IQ for the CaM were calculated with a 1:1 binding model. For odontocetes with increased EQ, the *K*d was 27.6 nM. Specially, with the EQ increasing, the *K*d values have a tendency to reduce, suggesting the interaction became weak with the EQ growth. For example, *K*d of the *T. truncates* with highest EQ (4.14) was 17.7 nM, whereas *K*d value increased 2.2 fold for the *P. macrocephalus* with lowest EQ (0.58), resulting from increased dissociation rate constants (*K*_off_). By contrast, the GST-IQ binding CaM could still be measured in the EQ decreased mysticetes, but *K*d value (mean 63.38 nM) increased dramatically due to increased *K*_off_ values. The result suggested that the affinity of this interaction in the odontocetes was stronger than for the mysticetes (Fig. 5c).

## Discussion

### Extensive adaptive evolution of cetacean *MCPH* genes

We systematically investigated the evolution of seven *MCPH* genes in cetaceans to explore the genetic basis of brain evolution in cetaceans. *MCPH1* genes appeared to be a pseudogene because premature stop codons and frameshift mutations were identified in three cetacean lineages. Evidence of pseudogene in *MCPH1* gene was also identified in other seven cetacean species by [19]. However, the sequences of the other six genes were intact. Strong evidence of positive selection was identified by site-specific modeling for four of six intact *MCPH* genes: *WDR62*, *CDK5RAP2*, *CEP152*, and *ASPM*. A total of 36 robust candidate sites under selection were identified by two ML methods (PAML and REL): six at *WDR62*, 16 at *CDK5RAP2*, six at *CEP152*, and eight at *ASPM*. Of these sites, 88.89% (32 / 36) were categorized as radical changes under positive selection at the protein-level. Notably, almost all of the radical amino acid changes subjected to positive selection were localized within or near the functional region of the predicted 3D structures of the four *MCPH* genes. For example, up to 87.50% positively selected sites (7 / 8) at *ASPM* were localized in the two putative key functional domains (i.e. calmodulin binding IQ motifs and microtubules domain), which play an essential role in orientation of mitotic spindles during embryonic neurogenesis. It has been reported that mutations in these putative functional domains would cause *MCPH* in humans [47, 48]. For *CDK5RAP2*, 50% (8 / 16) of sites under positive selection scattered over three predicated functional regions are known to play a role in the cohesion and condensation of chromosomes during mitosis [14].

According to ancestral state reconstruction of cetacean EQs [6, 49], almost all odontocetes have increased EQs whereas mysticetes have reduced EQ compared to their ancestor. Lineage-specific selection analyses found extensive positive selection at *CDK5RAP2* along cetacean lineages from the ancestor of cetaceans to descendant lineages, especially in lineages with both increased and decreased EQs. For *ASPM*, significant signs of positive selection were mainly determined in odontocete lineages with expanded EQ whereas the minke whale with contracted EQ was found to be under positive selection although none of the positively selected sites were identified. In contrast, only odontocetes with expanded EQ were subject to positive selection at *WDR62*. Such extensive adaptive evolution of *MCPH* genes in cetaceans may be well matched with complex evolution of their brain size, including EQ expansion in toothed whales and EQ reduction in baleen whales.

### Significant association between brain phenotype and *MCPH* gene evolution

Statistical association between selection on a functional gene and changes in phenotype are an important indication for exploring the genetic basis of adaptive phenotypes [21, 48]. Regressions revealed a significant positive association between relative brain size (EQ) and evolutionary rates for *ASPM* and *CDK5RAP2* but not for *WDR62* and *CEP152* (Fig. 3). However, both absolute brain mass and absolute body mass were not related to selection rates of the four positively selected *MCPH* genes. By contrast, *CDK5RAP2* and *ASPM* are related to neonatal brain mass in anthropoid primates whereas no association was found between these genes and EQ or adult brain size [16]. The discrepancy between anthropoid primates and cetaceans may be the result of different evolutionary patterns in brain size enlargement, although relaxed constraints on brain-body allometry were examined in both groups. Primates have a directional trend in brain mass expansion but not body mass, leading to a wide pattern of EQ expansion through primate evolution. However, cetacean EQ changes are associated with body mass [49]. For example, the reduced EQ in mysticetes was mainly driven by a high rate of body mass enlargement whereas the EQ increase in odontocetes was due to body mass decrease at the origin of odontocetes according to ancestral state reconstruction. Body mass changes are a predominant influence in cetacean EQ evolution [6, 49]. Despite large differences in EQ, mysticetes and odontocetes evolved with similar patterns of brain mass that were generally increased over time.

Although odontocetes have a generally increased EQ over time, there are some exceptions. It is well known that *P. macrocephalus* has the largest absolute brain mass (up to 10 kg) among animals but the smallest EQ. (0.58) among extant odontocetes due to its large body mass (more than 35,632 kg) [36]. *P. macrocephalus* had the second EQ reduction across cetacean lineages due to its 107-fold increase in body mass and only 6.5-fold increase in brain mass compared to their common ancestor [49]. Thus, we performed the association analyses after removing the sperm whale. The result revealed that *ASPM* and *CDK5RAP2* remain significantly related to EQ (*ASPM*: *R* ^2^ = 0.409, *P* = 0.028; *CDK5RAP2*: *R* ^2^ = 0.477, *P* = 0.016). The same pattern was also found after the removal of any one single species of odontocetes. This suggested that *P. macrocephalus* was not an outlier in the relationship between the genes’ evolutionary rates and EQ. However, when *P. macrocephalus* was excluded, a marginally negative relationship was identified between *ASPM* and absolute body mass (*R* ^2^ = 0.316, *P* = 0.053). This pattern was not found after any other single odontocete species was excluded. Thus, the sperm whale appeared to be an outlier in the association between the selection and body mass at the *ASPM*, which might attribute to its largest body mass. Additionally, bowhead whale *B. mysticetus* is another species with reduced EQ used in our association analysis. When the two species with reduced EQ. (*P. macrocephalus* and *B. mysticetus*) were excluded, *ASPM* is still significantly related to EQ (*R* ^2^ = 0.557, *P* = 0.013), suggesting the both species are not outliers. These results further corroborated our previous work that selection on *ASPM* may contribute to relative brain size enlargement during cetacean evolution [20]. Only two species with reduced EQ were used in our study and we should test whether cetaceans have a similar pattern to primates regarding *ASPM* and increasing and decreasing EQ when more data becomes available.

Notably, no association was found in the *ASPM* gene when only the two exons (approx. 60% of the transcribed *ASPM* protein) were examined in our previous study [20], which was questioned by Montgomery et al. [21]. However, when the 22 exons, accounting for 90.34% of the transcribed *ASPM* protein, were used in this study, it was striking and interesting to find a significant association between root-to-tip ω of *ASPM* and cetacean EQ (*R* ^2^ = 0.304, *P* = 0.046, Fig. 2). Therefore, it is best to use the complete CDS to explore gene-phenotype associations in the future.

### Is cetacean *ASPM* mainly linked to evolutionary increases in EQ?

*ASPM* plays a key role in mitotic spindle function including orientation of the cleavage plane [15]. To execute this function, *ASPM* must conjunct with CaM because *ASPM* is not detected at meiotic and mitotic spindles after RNAi of CaM [50]. Thus, it was suggested that CaM is needed for the localization of *ASPM*. More importantly, previous functional assays have proved that a minimal region of *ASPM*, such as the first IQ motif, can be sufficient for CaM binding [50]. Here, we examined binding affinities between CaM and the 23rd IQ (when the *T. truncates* as reference) of *ASPM* including one positively selected site in cetacean lineages to test whether there is a functional divergence between *ASPM* genes of EQ enlarged and contracted species.

The GST pull-down assay displayed that toothed species with the highest EQs had a strong effect on CaM binding to this IQ, whereas no such effect was found in baleen whales with decreased EQ (except for *B. acutorostrata*). The same result was found when the pull-down assay was repeated two times. *B. acutorostrata* is a special case for the pull-down assay of the baleen whales, likely because a significant sign of positive selection identified in this species but not in other baleen species at *ASPM*. In addition, *B. acutorostrata* is the smallest among the baleen whales with an average body length of 6.7–7.3 m and body mass of 11, 000 kg, similar to species of toothed whales. Specially, nearly significant negative association was identified between *ASPM* evolution and body mass (*P* = 0.052) in our association analysis when *P. macrocephalus* was excluded. Accordingly, the anomalous pattern in *B. acutorostrata* may be attributable it having the smallest body mass in the mysticetes.

Next, we quantitatively analyzed the binding affinities using BLI that revealed this IQ did bind to CaM in odontocetes with EQ expansion and mysticetes with EQ contraction. However, it was noted that the dissociation constants of mysticetes (mean 63.38 nM) were significantly higher than that of odontocetes (mean 27.6 nM), suggesting that the binding affinity of odontocetes with EQ expansion was stronger than for mysticetes. Notably, when only odontocetes were considered, there is a tendency that the *K*d values decrease with EQ increasing, suggesting the binding affinity of CaM and GST-IQ greatly increase with EQ increasing (Fig. 5). Such discrepancy of binding affinities of mysticetes between the GST pull down experiment and BLI analysis may be due to its weak binding affinity making it easy to wash out in the pull down experiment. Of course, only one IQ motif was detected in our study, the complete CDS should be tested when fresh tissue is available. Collectively, our functional assay further supported that cetacean *ASPM* was mainly linked to brain size expansion, which was contrasted with the finding in primates that *ASPM* evolution was related to both increase and decrease EQ [18].

### Molecular evidence to support social brain hypothesis in cetaceans

Field research shows that cetaceans, and particularly delphinids, live in large complex groups with highly differentiated relationships [51]. In such groups, cetaceans must identify their long-term bonds and higher-order alliances, and communicate, collaborate and compete among group members [12, 52]. It is widely accepted that brain size expansion in cetaceans is driven by complex social forces and cognitive demands for living in complex social groups [12, 52]. The ‘social brain hypothesis’ proposes that species living in a complex social group must manage a wide variety of information relevant to social living [8, 10, 53]. This hypothesis is supported by the correlation between high-level encephalization and sociality (particularly for stable groups) in mammalian species [54]. A positive relationship was found between the relative brain (or neocortex) size and group size in delphinids [13, 55], suggesting that relative brain size in delphinids enlarged in order to respond to cognitive demands, social complexity and group size. Similar results have been found in haplorhine primates [10, 56]. Group size is a proxy for social complexity, although it is not the driver of brain evolution [9, 53, 56].

In order to test whether the ‘social brain hypothesis’ is supported at the molecular level in cetaceans, we examined the association between positive selection on four *MCPH* genes and mean group size as summarized by May-Collado et al. [46]. Significant positive associations between evolutionary rate and mean group size were found for *ASPM* and *CDK5RAP2*. When only cetacean species with increased EQ were considered, a significant positive relationship remained for *CEP152* (*R* ^2^ = 0.519, *P* = 0.007). These findings confirm that cetaceans evolved large brains to manage their unusually complex social systems. Although mean group size is a crude measure of social complexity, group size data is relatively easy to obtain for wild mammals. Relationships between *MCPH* gene evolution and other ecological factors of sociality such as pair bonding, activity patterns and diet should be examined in cetaceans to further consolidate the social brain hypothesis at the molecular level.

## Conclusions

Cetaceans evolved a dramatic brain size expansion but their body evolved in complex pattern, leading to odontocetes with increased EQ and mysticetes with decreased EQ. We comprehensively investigated seven *MCPH* genes associated with brain size development in representative cetacean lineages. Significant positive selection was examined at the four *MCPH* genes, special for *ASPM* and *CDK5RAP2* genes, selection identified along lineages with both increased and decreased EQ. The result is well matched with cetacean complex brain size evolution. Association analyses showed that *CDK5RAP2* and *ASPM* evolutionary rate (ω) were significantly related to EQ, suggesting that these two genes may have contributed to EQ expansion in cetaceans. This suggestion was further indicated by our preliminary function test that *ASPM* might be mainly linked to evolutionary increases in EQ. In addition, a positive association was determined between evolution rate of *ASPM* and *CDK5RAP2* and mean group size, which is consistent with ‘social brain hypothesis’ that that cetaceans evolved large brains to manage complex social systems.

## Additional files

Additional file 1: Table S1. Sequence information of seven MCPH genes across the phylogeny of Cetartiodactyla used in this study. **Table S2.** Results for site model and free-ratio model analysis at the six MCPH genes using the gene tree and species tree. **Table S3.** Morphological variables of cetacean brain used in regression analyses. **Table S4.** Amino acid sites under positive selection identified by maximum likelihood (ML) methods and TreeSAAP. **Table S5.** Regression analyses between the root-to-tip ω and body mass, body mass, and EQ across cetacean lineages. **Table S6.** Phylogenetically controlled regression analyses between the root-to-tip ω and mean group size. (DOC 239 kb)

Additional file 2: Supplementary methods and results. **Figure S1.** Phylogeny of cetaceans based on ML and BI best topology; number above branches show bootstrap support and posterior probability value above 0.50. (ZIP 759 kb)

Additional file 3: Alignment sequences of seven MCPH genes used for this study. (DOCX 60 kb)

## Abbreviations

3D: three-dimensional
BEB: Bayes Empirical Bayes
BI: Bayesian inference
BLI: biolayer interferometry
CaM: calmodulin
CDS: coding sequence
*d*_N_: nonsynonymous substitution
*d*_S_: synonymous substitution
EQ: encephalization quotients
FDR: false discovery rate
FEL: fixed effects likelihood
*K*d: dissociation constants
*K*off: dissociation rate constants
*K*on: association rate constants
LRT: likelihood ratio test
M0: one-ratio model
M1: free-ratio model
Ma: million years ago
*MCPH*: microcephaly
ML: maximum likelihood
PGLS: phylogenetic generalized least squares
