# Supplementary material for: Genetic basis of brain size evolution in cetaceans: insights from adaptive evolution of seven primary microcephaly (MCPH) genes
Source: BMC Evol Biol. 2017 Aug 29;17:206. doi: 10.1186/s12862-017-1051-7 (PMC5576371; doi:10.1186/s12862-017-1051-7)
Supplement: Supplementary file 1 — Sequence information of seven MCPH genes across the phylogeny of Cetartiodactyla used in this study. Table S2. Results for site model and free-ratio model analysis at the six MCPH genes using the gene tree and species tree. Table S3. Morphological variables of cetacean brain used in regression analyses. Table S4. Amino acid sites under positive selection identified by maximum likelihood (ML) methods and TreeSAAP. Table S5. Regression analyses between the root-to-tip ω and body mass, body mass, and EQ across cetacean lineages. Table S6. Phylogenetically controlled regression analyses between the root-to-tip ω and mean group size. (DOC 239 kb) [file 12862_2017_1051_MOESM1_ESM.doc]

**Table S1 Sequence information of seven *MCPH* genes across the phylogeny of Cetartiodactyla used in this study**

| Classification | Species name | Common name | Accession numbers | | | | | | | |
| --- | --- | --- | --- | --- | --- | --- | --- | --- | --- | --- |
|  |  |  | MCPH1 | WDR62 | CDK5RAP2 | | CEP152 | ASPM | CENPJ | STIL |
| Family  Hippopotamidae | *Hippopotamus amphibius* | Hippopotamus | HM771327 | - | - | - | | HQ141114 | - | This study |
| Family Bovidae | *Bos taurus* | Cow | ENSBTAT00000031604 | ENSBTAT00000019966 | ENSBTAT00000008586 | ENSBTAT00000015487 | | ENSBTAT00000010340 | ENSBTAT00000022667 | ENSBTAG00000017844 |
| Suborder Odontoceti | | | | | | | | | | |
| Family  Delphinidae | *Delphinus capensis* | Long-beaked commom dolphin | This study | This study | This study | This study | | This study | This study | This study |
|  | *Grampus griseus* | Risso's dolphin | This study | This study | This study | This study | | This study | This study | This study |
|  | *Stenella coeruleoalba* | Striped dolphin | This study | This study | This study | This study | | This study | This study | This study |
|  | *Sousa chinensis* | Chinese white dolphin | This study | This study | This study | This study | | This study | This study | This study |
|  | *Tursiops aduncus* | Indo-pacific bottlenose dolphin | This study | This study | This study | This study | | This study | This study | This study |
|  | *T. truncatus* | Bottlenose dolphin | This study | This study | This study | This study | | This study | This study | This study |
|  | *Orcinus orca* | Killer whale | XM_012537135 | XM_012538380 | XM_004269283 | XM_012537281 | | XM_012538805 | XM_012537420 | XM_004273897 |
| Family  Phocoenidae | *Neophocaena phocaenoides* | Finless porpoise | This study | This study | This study | This study | | This study | This study | This study |
| Family  Monodontidae | *Delphinapterus leucas* | Beluga whale | This study | This study | This study | This study | | This study | This study | This study |
| Family Lipotidae | *Lipotes vexillifer* | Yangtze River Dolphin | This study | This study | This study | This study | | This study | This study | This study |
| Family Ziphiidae | *Mesoplodon densirostris* | Blainville's beaked whale | This study | This study | This study | This study | | This study | This study | This study |
| Family  Physeteridae | *Kogia sima* | Dwarf sperm whale | This study | This study | This study | This study | | This study | This study | This study |
|  | *Physeter macrocephalus* | Sperm whale | XM_007114976 | XM_007101100 | XM_007123640 | XM_007121287 | | XM_007106790 | XM_007114191 | XM_007128448 |
| Suborder Mysticeti | | | | | | | | | | |
| Family  Balaenopteridae | *Balaenoptera acutorostrata* | Common minke whale | This study | This study | This study | This study | | This study | This study | This study |
| Family  Balaenoptiidae | *B.omurai* | Omura's whale | This study | This study | This study | This study | | This study | This study | This study |
| Family  Balaenidae | *B.mysticetus* | Bowhead whale | 15996 bmy_22635T0 | 8281 bmy_05918T0 | 4469 bmy_17483T0 | 5218 bmy_17798T0 | | 5369 bmy_18025T0 | 18040 bmy_11019T0 | 20071 bmy_12717T0 |

All the newly obtained sequences were deposited in GenBank, accession numbers: KY011963- KY012055

**Table S2 Results for site model and free-ratio model analysis at the six MCPH genes using the** gene tree and “species tree”

| **Genes** | **models** | **-lnL** | **models compared** | **2ΔLnL** | **d.f.** | **p-values** | **parameter estimates** | **branch under positive selection** |
| --- | --- | --- | --- | --- | --- | --- | --- | --- |
| **Site models** |  |  |  |  |  |  |  |  |
| **ASPM (species tree)** | M7 | 20714.924 | M7 versus M8 | 28.432 | 2 | <0.001 |  | ---- |
|  | M8 | 20700.708 |  |  |  |  | ω=5.236 |  |
|  | M8a | 20714.804 | M8a versus M8 | 28.191 | 1 | <0.001 |  |  |
| **ASPM**  **(gene tree)** | M7 | 20680.483 | M7 versus M8 | 76.920 | 2 | <0.001 |  | ---- |
|  | M8 | 20642.023 |  |  |  |  | ω=3.768 |  |
|  | M8a | 20653.281 | M8a versus M8 | 22.516 | 1 | <0.001 |  |  |
| **CENPJ (species tree)** | M7 | 7086.473 | M7 versus M8 | 0.866 | 2 | 0.649 |  | ---- |
|  | M8 | 7086.040 |  |  |  |  | ω=1.593 |  |
|  | M8a | 7086.280 | M8a versus M8 | 0.488 | 1 | 0.485 |  |  |
| **CENPJ**  **(gene tree)** | M7 | 7081.632 | M7 versus M8 | 0.945 | 2 | 0.623 |  | ---- |
|  | M8 | 7081.160 |  |  |  |  | ω=1.216 |  |
|  | M8a | 7081.315 | M8a versus M8 | 0.311 | 1 | 0.577 |  |  |
| **STIL**  **(species tree)** | M7 | 6173.287 | M7 versus M8 | 1.700 | 2 | 0.427 |  | ---- |
|  | M8 | 6172.437 |  |  |  |  | ω=4.921 |  |
|  | M8a | 6173.275 | M8a versus M8 | 1.677 | 1 | 0.195 |  |  |
| **STIL**  **(gene tree)** | M7 | 6170.080 | M7 versus M8 | 1.910 | 2 | 0.385 |  | ---- |
|  | M8 | 6169.125 |  |  |  |  | ω=5.700 |  |
|  | M8a | 6170.082 | M8a versus M8 | 1.914 | 1 | 0.167 |  |  |
| **WDR62**  **(species tree)** | M7 | 7703.257 | M7 versus M8 | 24.872 | 2 | <0.001 |  | ---- |
|  | M8 | 7690.821 |  |  |  |  | ω=10.203 |  |
|  | M8a | 7703.049 | M8a versus M8 | 24.456 | 1 | <0.001 |  |  |
| **WDR62**  **(gene tree)** | M7 | 7698.629 | M7 versus M8 | 22.500 | 2 | <0.001 |  | ---- |
|  | M8 | 7687.379 |  |  |  |  | ω=9.773 |  |
|  | M8a | 7698.459 | M8a versus M8 | 22.160 | 1 | <0.001 |  |  |
| **CEP152**  **(species tree)** | M7 | 9949.080 | M7 versus M8 | 19.662 | 2 | <0.001 |  | ---- |
|  | M8 | 9939.249 |  |  |  |  | ω=7.312 |  |
|  | M8a | 9948.922 | M8a versus M8 | 19.347 | 1 | <0.001 |  |  |
| **CEP152**  **(gene tree)** | M7 | 9937.723 | M7 versus M8 | 15.548 | 2 | <0.001 |  | ---- |
|  | M8 | 9929.949 |  |  |  |  | ω=5.317 |  |
|  | M8a | 9937.498 | M8a versus M8 | 15.098 | 1 | <0.001 |  |  |
| **Branch model** | | | | | | | | |
| **ASPM**  **(species tree)** | M0:one-ratio | 27440.959 | M0 versus M1 | 73.957 | 33 | <0.001 | ω=0.420 |  |
|  | M1:free-ratio | 27403.981 |  |  |  |  | ω variation for each branch | Last common ancestors of the *Lipotes vexillifer*  (ω=1.1286 20.4/6.8)  Last common ancestors of Delphinida  (ω=1.4003 12.2/3.3)  Last common ancestors of (*Neophocaena phocaenoides*+ *Delphinapterus leucas*) (ω=1.0096 8.1/3.0) |
| **ASPM**  **(gene tree)** | M0:one-ratio | 27373.605 | M0 versus M1 | 75.097 | 33 | <0.001 | ω=0.41862 |  |
|  | M1:free-ratio | 27336.056 |  |  |  |  | ω variation for each branch | Last common ancestors of the *Lipotes vexillifer*  (ω=1.1301 20.4/6.8)  Last common ancestors of Delphinida (1.4003 12.2/3.3)  Last common ancestors of (*Neophocaena phocaenoides*+ *Delphinapterus leucas*) (ω=1.0118 8.1/3.0) |
| **CENPJ**  **(species tree)** | M0:one-ratio | 8398.170 | M0 versus M1 | 26.262 | 31 | 0.709 | ω= 0.442 |  |
|  | M1:free-ratio | 8385.040 |  |  |  |  | ω variation for each branch |  |
| **CENPJ**  **(gene tree)** | M0:one-ratio | 8376.785 | M0 versus M1 | 27.412 | 31 | 0.741 | ω=0.44828 |  |
|  | M1:free-ratio | 8390.491 |  |  |  |  | ω variation for each branch |  |
| **STIL**  **(species tree)** | M0:one-ratio | 7703.648 |  |  |  |  | ω=0.397 |  |
|  | M1:free-ratio | 7685.495 | M0 versus M1 | 36.305 | 33 | 0.317 | ω variation for each branch |  |
| **STIL**  **(gene tree)** | M0:one-ratio | 9027.159 |  |  |  |  | ω=0.300 |  |
|  | M1:free-ratio | 9005.050 | M0 versus M1 | 44.218 | 31 | 0.058 | ω variation for each branch |  |
| **WDR62**  **(species tree)** | M0:one-ratio | 9033.990 |  |  |  |  | ω= 0.303 |  |
|  | M1:free-ratio | 9013.151 | M0 versus M1 | 41.678 | 31 | 0.095 | ω variation for each branch |  |
| **WDR62**  **(gene tree)** | M0:one-ratio | 9027.159 |  |  |  |  | ω=0.300 |  |
|  | M1:free-ratio | 9005.050 | M0 versus M1 | 44.218 | 31 | 0.058 | ω variation for each branch |  |
| **CEP152**  **(species tree)** | M0:one-ratio | 11505.268 |  |  |  |  | ω=0.468 |  |
|  | M1:free-ratio | 11485.975 | M0 versus M1 | 38.587 | 31 | 0.164 | ω variation for each branch |  |
| **CEP152**  **(gene tree)** | M0:one-ratio | 11489.746 |  |  |  |  | ω= 0.467 |  |
|  | M1:free-ratio | 11470.200 | M0 versus M1 | 39.092 | 31 | 0.151 | ω variation for each branch |  |

Species tree was reconstructed using the maximum-likelihood (ML) and Bayesian inference.

**Table S3** **Morphological variables of cetacean brain and mean group size used in regression analyses**

| **Speices** | **Brain mass (g)** | **Body mass (g)** | **References** | **EQ^1^** | **Mean Group size** | **References** |
| --- | --- | --- | --- | --- | --- | --- |
|
| *Tursiops truncatus* | 1759.2 | 206823.8 | Pilleri and Gihr 1970 | 4.02 | 92.2 | May-Collado et al. 2007 |
| *Grampus griseus* | 2384.4 | 319974 | Marino et al. 2004 | 4.07 | 63 | May-Collado et al. 2007 |
| *Stenella coeruleoalba* | 820 | 56300 | Schwerdtfeger et al. 1984 | 4.48 | 302 | May-Collado et al. 2007 |
| *Delphinapterus leucas* | 2083 | 636000 | Ridgway et al. 1984 | 2.25 | 32.9 | May-Collado et al. 2007 |
| *Lipotes vexillifer* | 510 | 82000 | Marino et al. 2004 | 2.17 | 3.4 | May-Collado et al. 2007 |
| *Mesoplodon densirostris* | 1457.5 | 763334.6 | Marino et al. 2004 | 1.39 | 3.7 | May-Collado et al. 2007 |
| *Kogia sima* | 621.5 | 167958.3 | Marino et al. 2004 | 1.63 | 1.87 | May-Collado et al. 2007 |
| *Neophocaena phocaenoides* | 468 | 32400 | Schwerdtfeger et al. 1984 | 3.71 | 3 | May-Collado et al.2007 |
| *Physeter catodon* | 7999 | 35632154 | Pilleri and Gihr 1970 | 0.58 | 22.1 | May-Collado et al. 2007 |
| *Orcinus orca* | 5028 | 1953201.4 | Pilleri and Gihr 1970 | 2.56 | 12 | May-Collado et al. 2007 |
| *Balaena mysticetus* | 2738 | 91000000 | Stephan et al. 1981 | 0.106 | 1 | May-Collado et al. 2007 |
| *Delphinus capensis* | - | - | - | - | 411.69 | May-Collado et al. 2007 |
| *Sousa chinensis* | - | - | - | - | 14.9 | May-Collado et al. 2007 |

^1^ EQ = brain weight / 0.12 (body weight)^0.67^

**Table S4** **Amino acid sites under positive selection identified by maximum likelihood (ML) methods and TreeSAAP**

| **Gene** | **Amino acid position** | **Maximum Likelihood Method TreeSAAP ^c^** | | | |
| --- | --- | --- | --- | --- | --- |
|  |  | **PAML M8 (*p* > 0.8) ^a^** | **FEL**  **(*p* < 0.2) ^b^** | **Radical Changes in Amino acid Properties** | **Total ^d^** |
| WDR62 | 262 | 0.995 | 0.114 | *Ns Br c Ca h pH_i_ Hnc V^0^ p* *Et* *Esm* | 11 (4) |
|  | 458 | 0.994 | 0.110 | *pH_i_ F α_c_ α_m_* | 4 (-) |
|  | 479 | 0.927 | 0.152 | *pH_i_* | 1 (-) |
|  | 972 | 0.947 | 0.131 | *Pα* | 1 (1) |
|  | 1008 | 0.989 | 0.029 | *Ns Br R_f_ Pc pK’ h El F Pr p* *Ra Hp Ht Et P* | 15 (2) |
|  | 1133 | 0.944 | 0.068 | *Esm* | 1 (-) |
| CDK5RAP2 | 23 | 0.913 | 0.118652 | *P_β_ Bl Pc F Ra P* | 6 (-) |
|  | 108 | 1.000 | 0.0000453349 | *Ns P_β_ R_f_ c El V^0^Pr p* *α_c_ Ht* | 10 (-) |
|  | 182 | 0.930 | 0.136353 | *pH_i_ Et* | 2 (-) |
|  | 201 | 0.967 | 0.093099 | *Pα Pc α_c_ Ht P* | 5 (1) |
|  | 283 | 0.931 | 0.185303 | — | — |
|  | 426 | 0.999 | 0.0643632 | *Pα Pc K^0^ pH_i_ α_c_ P* | 6 (2) |
|  | 445 | 0.856 | 0.197358 | *pH_i_ α_c_* | 2 (1) |
|  | 531 | 0.962 | 0.190062 | *Ns Br R_f_ c Ca h pH_i_ Hnc p* *Esm Et* | 11 (2) |
|  | 720 | 0.960 | 0.101684 | *K^0^ F α_c_ α_m_ Esm* | 5 (-) |
|  | 760 | 0.856 | 0.136792 | *α_c_ Ht* | 2 (-) |
|  | 787 | 0.947 | 0.168683 | *K^0^ α_c_ α_m_ Esm* | 4 (-) |
|  | 832 | 0.833 | 0.114707 | *Pc K^0^ pH_i_ α_m_ Ht* | 5 (0) |
|  | 849 | 0.910 | 0.193987 | *pH_i_ α_c_* | 2 (1) |
|  | 913 | 0.928 | 0.193987 | *Pr α_c_* | 2 (-) |
|  | 1166 | 0.928 | 0.19792 | *K^0^ F α_c_ α_m_* | 4 (-) |
|  | 1284 | 0.878 | 0.195115 | — | — |
| CEP152 | 79 | 0.903 | 0.189 | *E_sm_* | 1 (1) |
|  | 305 | 0.951 | 0.140 | *E_sm_* | 1 (1) |
|  | 510 | 0.971 | 0.069 | — | — |
|  | 1147 | 0.964 | 0.152 | *h* *p* | 2 (-) |
|  | 1163 | 0.918 | 0.100 | *N_s_* *B_r_* *pK'* *H_t_ K^0^**p* *P* *E_t_* *P Hp P_α_ R_a_* | 12 (2) |
|  | 1398 | 0.910 | 0.136 | — | — |
| ASPM | 69 | 0.807 | 0.114737 | *pHi* | 1 (-) |
|  | 347 | 0.823 | 0.180757 | *Ns Pc c pK’ Ca pHi F Mv Mw Hnc V^0^ µ Esm Et* | 14 (-) |
|  | 387 | 0.857 | 0.135695 | *αc αm* | 2 (-) |
|  | 692 | 0.996 | 0.116516 | *Pα P* | 2 (-) |
|  | 1311 | 0.889 | 0.107427 | *Ns Br c Ca h pHi Hnc p Esm Et* | 10 (5) |
|  | 1602 | 0.814 | 0.150999 | *P_β_* | 1 (-) |
|  | 1864 | 0.829 | 0.117554 | *Pα Ra* | 2 (1) |
|  | 2619 | 0.885 | 0.040559 | *Ns Bl R_f_ Pc F α_n_ P* | 7 (-) |

**Note**: Positively selected sites identified by at two ML methods are presented here.

^a^ Codons identified by M8 model in PAML using a Bayes Empirical Bayes (BEB) analysis with posterior probabilities ≥ 0.80.

^b^ Condons determined by FEL implemented in HYPHY with significance levels of 0.2.

^c^ TreeSAAP: radical changes in amino acid properties (chemical, structural, and other property changes) under category 6 and/or 7 and/or 8 are shown. Amino acid sites with greater than three radical changes in amino acid properties are marked with grey, such strong positive selection with *P* ≤ 0.05 are shown in underline, whereas those positively selected sites with no radical changes are indicated in “—”. Amino acid property symbols used: α− helical tendencies (*Pα*), average number of surrounding residues (*Ns*), β-structure tendencies (*P_β_*), bulkiness (*Bl*), buriedness (*Br*), composition (*c*), compressibility (*K^0^*)，chromatographic index (*R_f_*), coil tendencies (*Pc*), equilibrium constant for ionization of COOH (*pK’*), helical contact energy (*Ca*), Hydropathy (*h*), isoelectric point (*pH_i_*), long-range n.b. energy (*E_l_*), mean r.m.s. ﬂuctuat. displace. (*F*), molecular volume (*M_v_*), molecular weight (*M_w_*), normalized consensus hydrophobicity (*H_nc_*), partial speciﬁc volume (*V^0^*), polar requirement (*P_r_*), polarity (*p*), power to be – N-term α -helix (*α_n_*), power to be at C-terminus of the a-helix (*α_c_*), power to be in the middle of an a-helix (*α_m_*), refractive index (*µ*), solvent accessible reduction ratio (*R_a_*), surrounding hydrophobicity (*H_p_*), sh.- & med.-range n.b. energy (*E_sm_*), thermodyn. transfer hydrophob. (*H_t_*), total n.b. energy (*E_t_*), turn tendencies (*P*).

^d^ The “total” indicates the number of radical changes in amino acid properties and the sites under strong positive selection with *P* ≤ 0.05 showed in the parentheses.

**Table S5 Regression analyses between the root-to-tip ω and body mass, body mass, and EQ across cetacean lineages**

| **Gene** | **Traits** | ***R^2^*** | ***F*** | ***P*** | **λ** |
| --- | --- | --- | --- | --- | --- |
| *ASPM* | EQ | 0.304 | 5.373 | **0.046** | 1 |
|  | Brain mass | 0.005 | 0.041 | 0.844 | 0 |
|  | Body mass | 0.213 | 3.711 | 0.086 | 0 |
| *CDK5RAP2** | EQ | 0.521 | 11.870 | **0.007** | 0.953 |
|  | Brain mass | 1.18E-04 | 0.001 | 0.975 | 0 |
|  | Body mass | 0.149 | 2.752 | 0.132 | 0 |
| *WDR62* | EQ | 0.235 | 4.068 | 0.074 | 1 |
|  | Brain mass | 0.004 | 0.033 | 0.861 | 0 |
|  | Body mass | 0.040 | 1.413 | 0.265 | 0.963 |
| *CEP152* | EQ | 0.005 | 0.043 | 0.841 | 1 |
|  | Brain mass | 0.004 | 0.033 | 0.861 | 0 |
|  | Body mass | 0.002 | 0.014 | 0.909 | 0.931 |

Significant *P* values are highlighted in bold.

*For *CDK5RAP2*, no significant association between log (root-to-tip ω) and log (EQ) was determined by PGLS analysis (*R*^2^ = 0.105, *P* = 0.175) but a significant positive relationship was found by OLS (*R*^2^ = 0.521, *P* = 0.007). Thus, OLS result could be accepted whereas reject the PGLS analysis even if phylogenetic signal was determined at this gene.

**Table S6 Phylogenetically controlled regression analyses between the root-to-tip ω and mean group size**

| **Gene** | ***R*^2^** | ***F*** | ***P*** | ***λ*** |
| --- | --- | --- | --- | --- |
| *ASPM* | 0.267 | 5.361 | **0.041** | 0 |
| *CDK5RAP2* | 0.308 | 6.333 | **0.029** | 0 |
| *WDR62* | 0.036 | 0.413 | 0.534 | 0.683 |
| *CEP152* | 0.086 | 2.126 | 0.173 | 0.492 |

Significant *P* values are highlighted in bold.
