# Supplementary figures and images for: Genetic basis of brain size evolution in cetaceans: insights from adaptive evolution of seven primary microcephaly (MCPH) genes
Source: BMC Evol Biol. 2017 Aug 29;17:206. doi: 10.1186/s12862-017-1051-7 (PMC5576371; doi:10.1186/s12862-017-1051-7)

WDR62

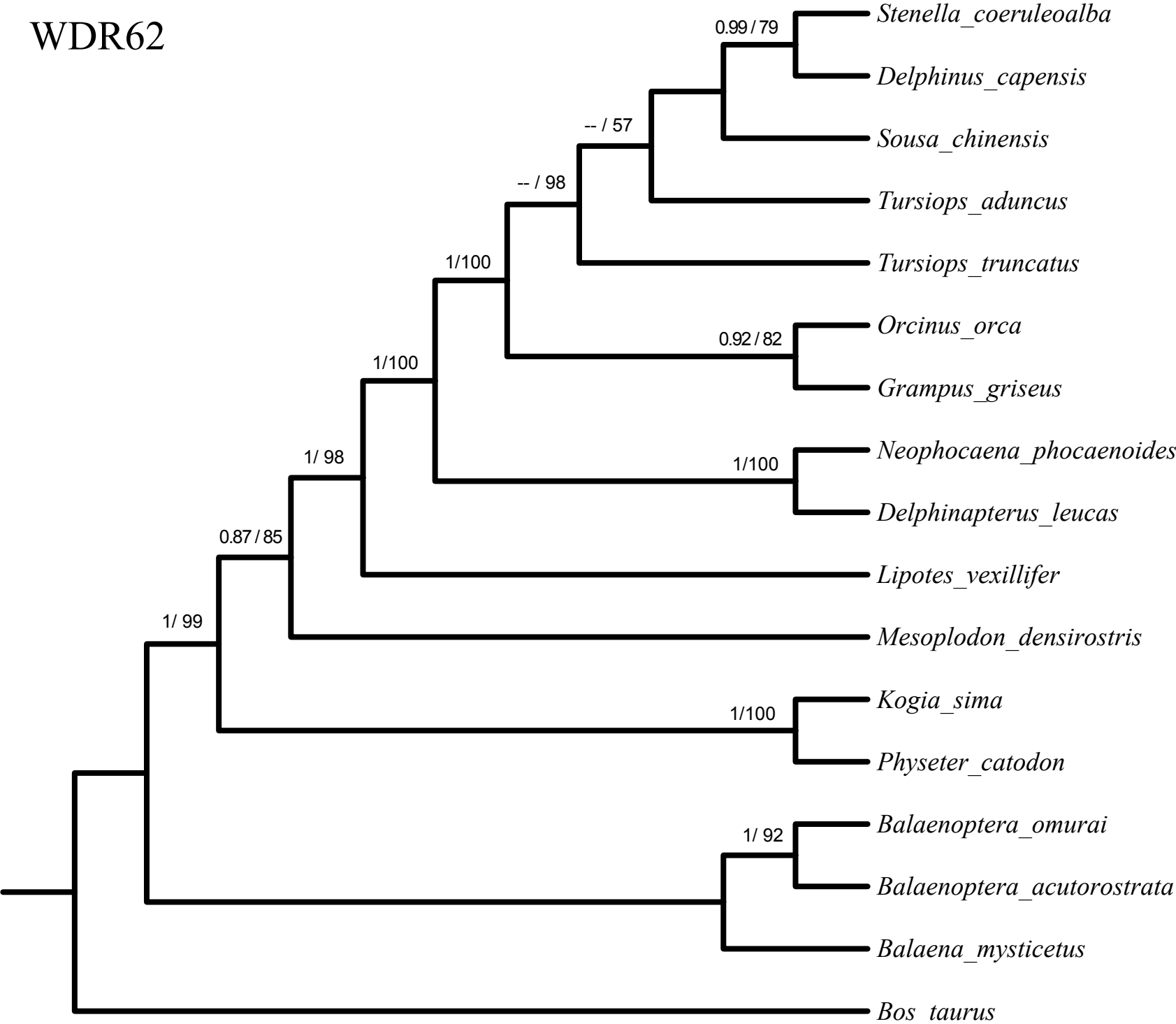

0.008

Supplement: Supplementary file 2 — Supplementary methods and results. Figure S1. Phylogeny of cetaceans based on ML and BI best topology; number above branches show bootstrap support and posterior probability value above 0.50. (ZIP 759 kb) [file 12862_2017_1051_MOESM2_ESM.zip › Fig. S1A Phylogeny of cetaceans at WDR62.PDF]

CDK5RAP2

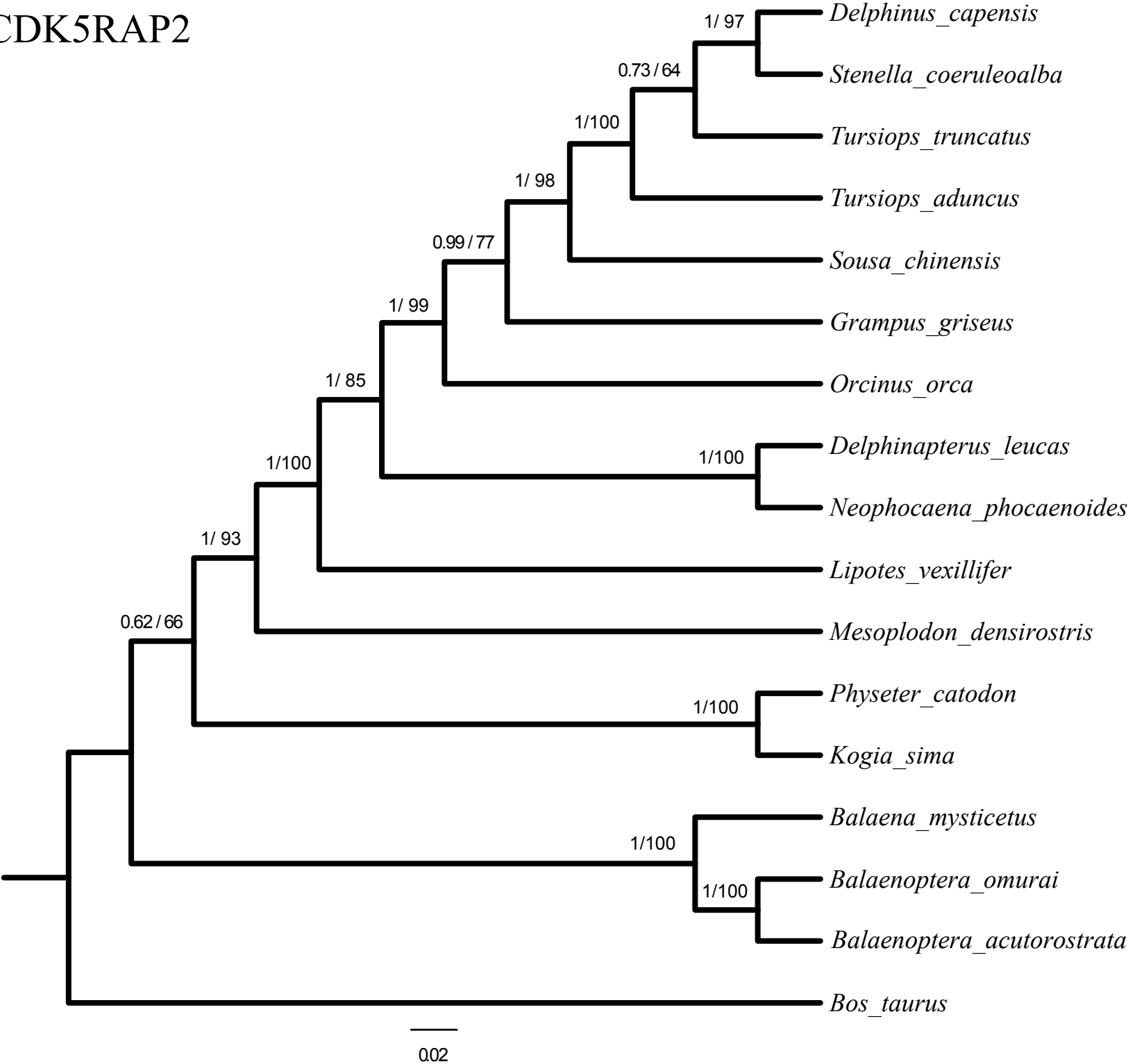

Supplement: Supplementary file 2 — Supplementary methods and results. Figure S1. Phylogeny of cetaceans based on ML and BI best topology; number above branches show bootstrap support and posterior probability value above 0.50. (ZIP 759 kb) [file 12862_2017_1051_MOESM2_ESM.zip › Fig. S1B Phylogeny of cetaceans at CDK5RAP2.PDF]

CEP152

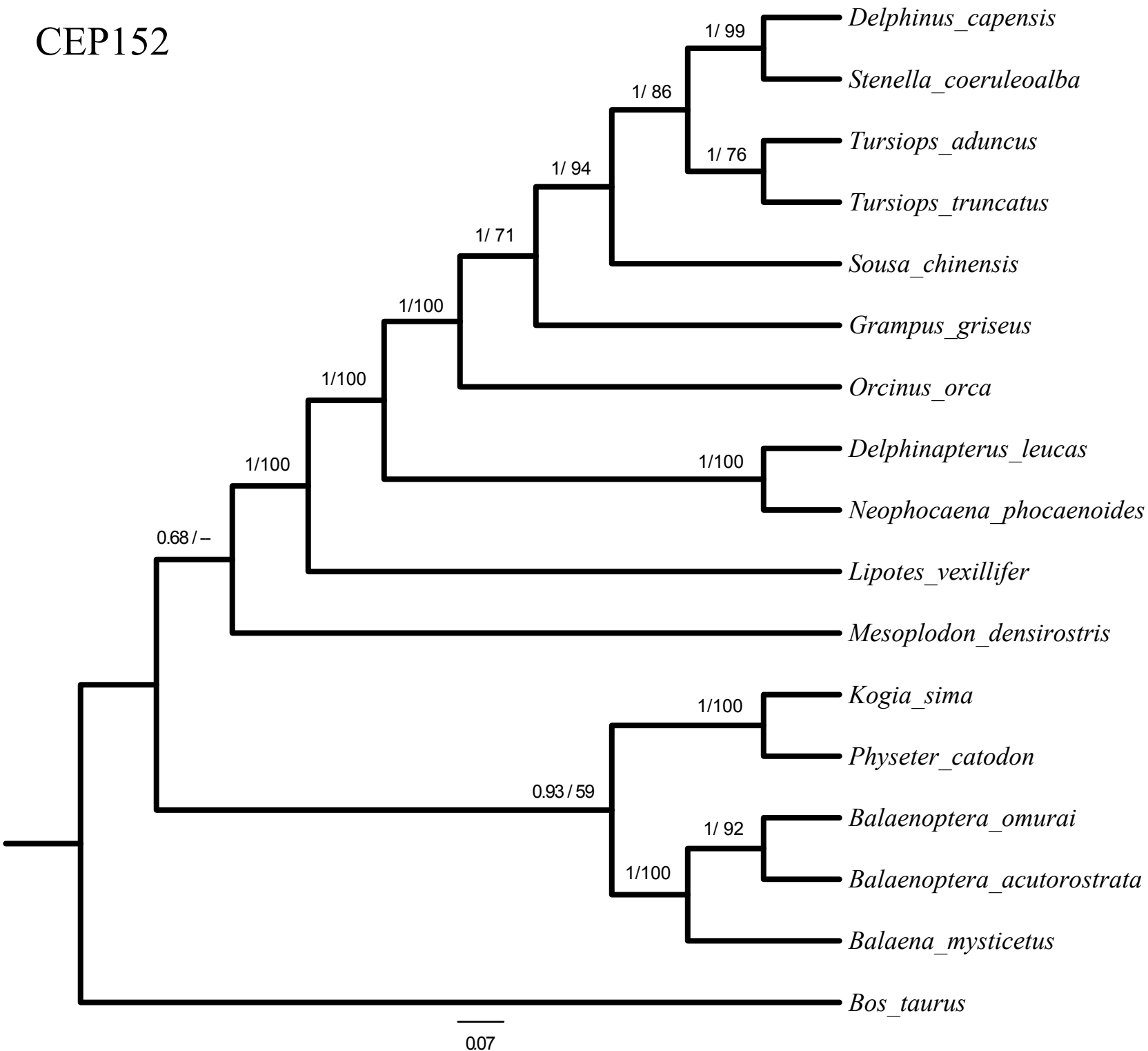

Supplement: Supplementary file 2 — Supplementary methods and results. Figure S1. Phylogeny of cetaceans based on ML and BI best topology; number above branches show bootstrap support and posterior probability value above 0.50. (ZIP 759 kb) [file 12862_2017_1051_MOESM2_ESM.zip › Fig. S1C Phylogeny of cetaceans at CEP152.PDF]

ASPM

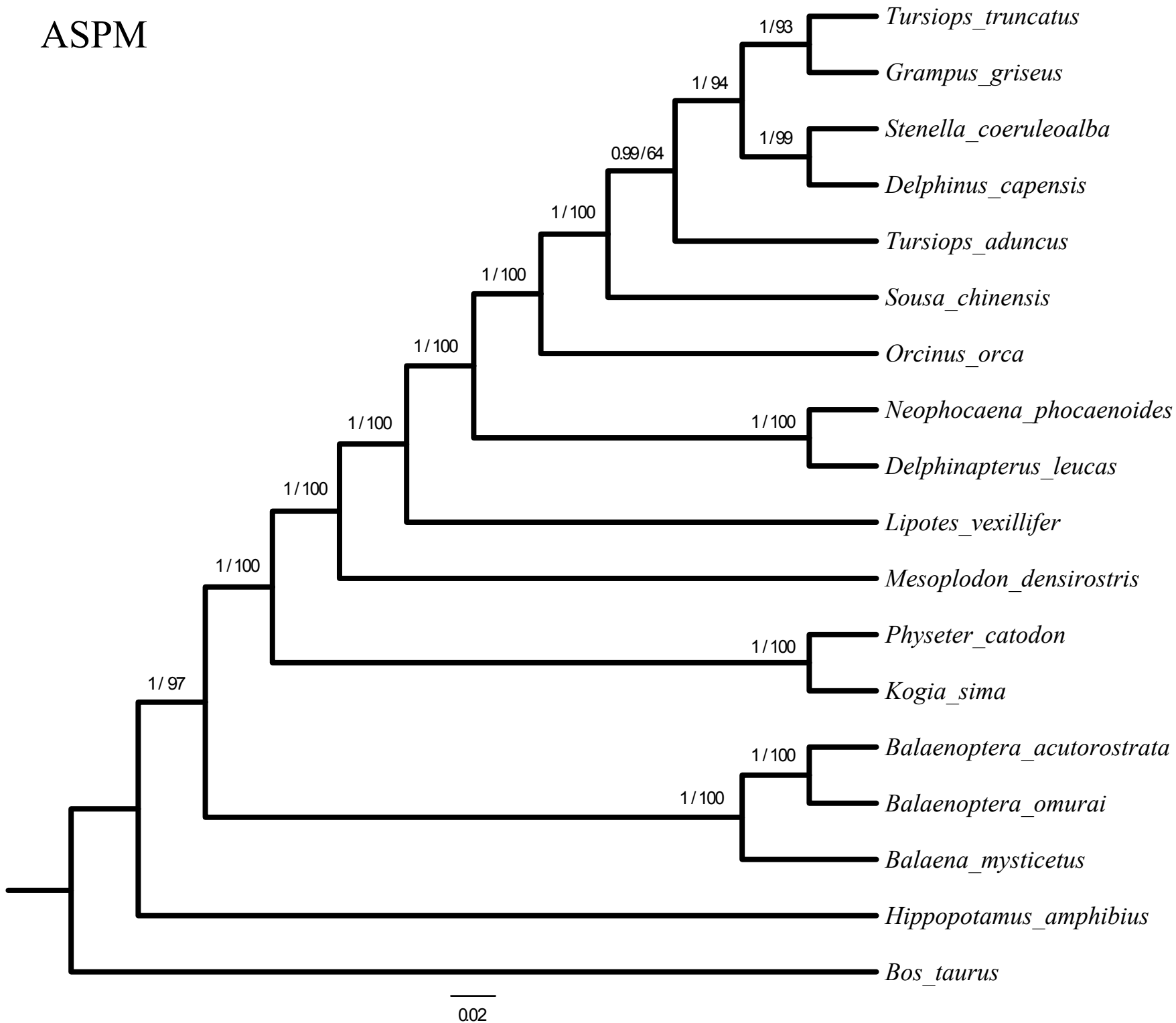

Supplement: Supplementary file 2 — Supplementary methods and results. Figure S1. Phylogeny of cetaceans based on ML and BI best topology; number above branches show bootstrap support and posterior probability value above 0.50. (ZIP 759 kb) [file 12862_2017_1051_MOESM2_ESM.zip › Fig. S1D Phylogeny of cetaceans at ASPM.PDF]

CENPJ

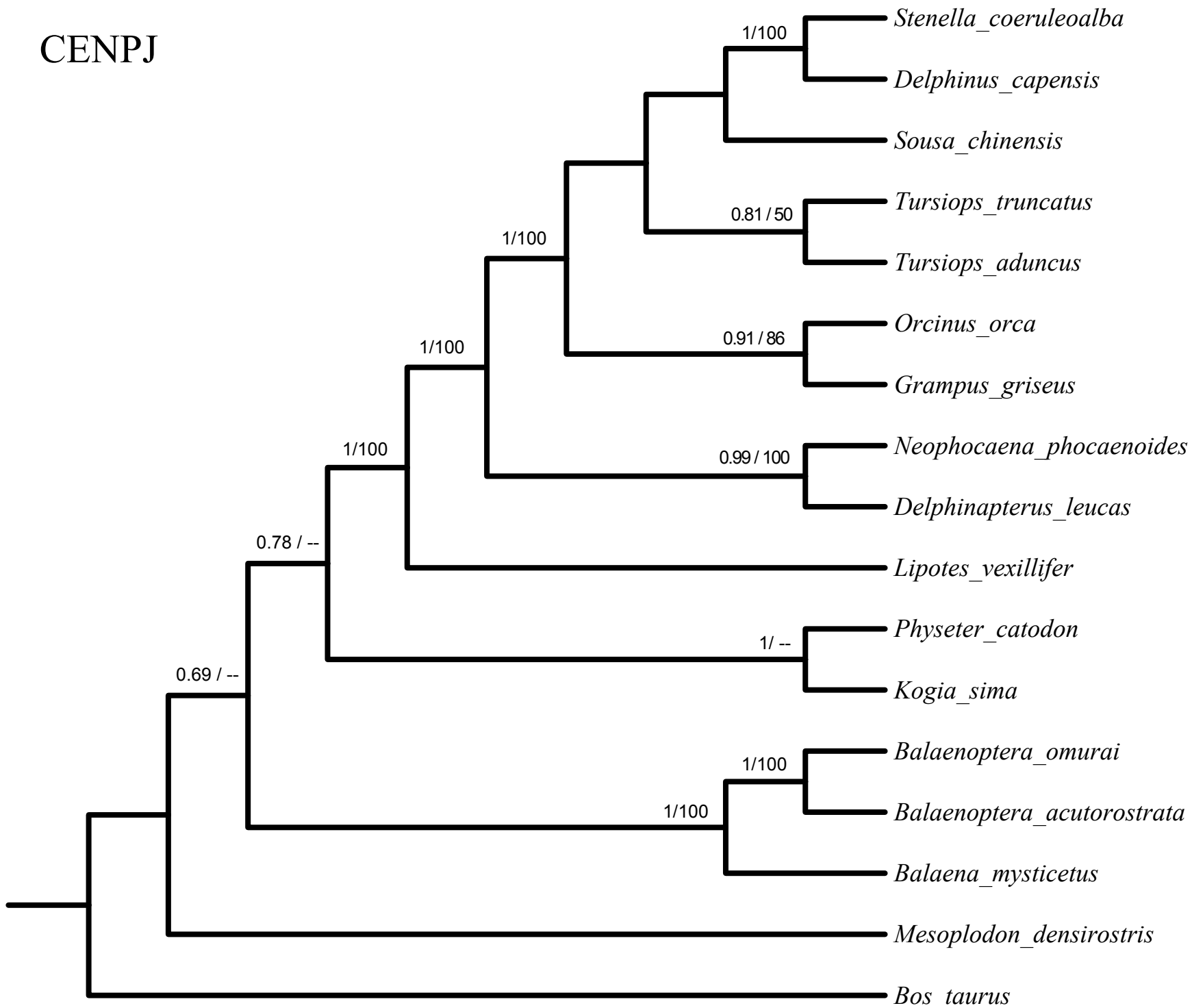

0.02

Supplement: Supplementary file 2 — Supplementary methods and results. Figure S1. Phylogeny of cetaceans based on ML and BI best topology; number above branches show bootstrap support and posterior probability value above 0.50. (ZIP 759 kb) [file 12862_2017_1051_MOESM2_ESM.zip › Fig. S1E Phylogeny of cetaceans at CENPJ.PDF]

STIL

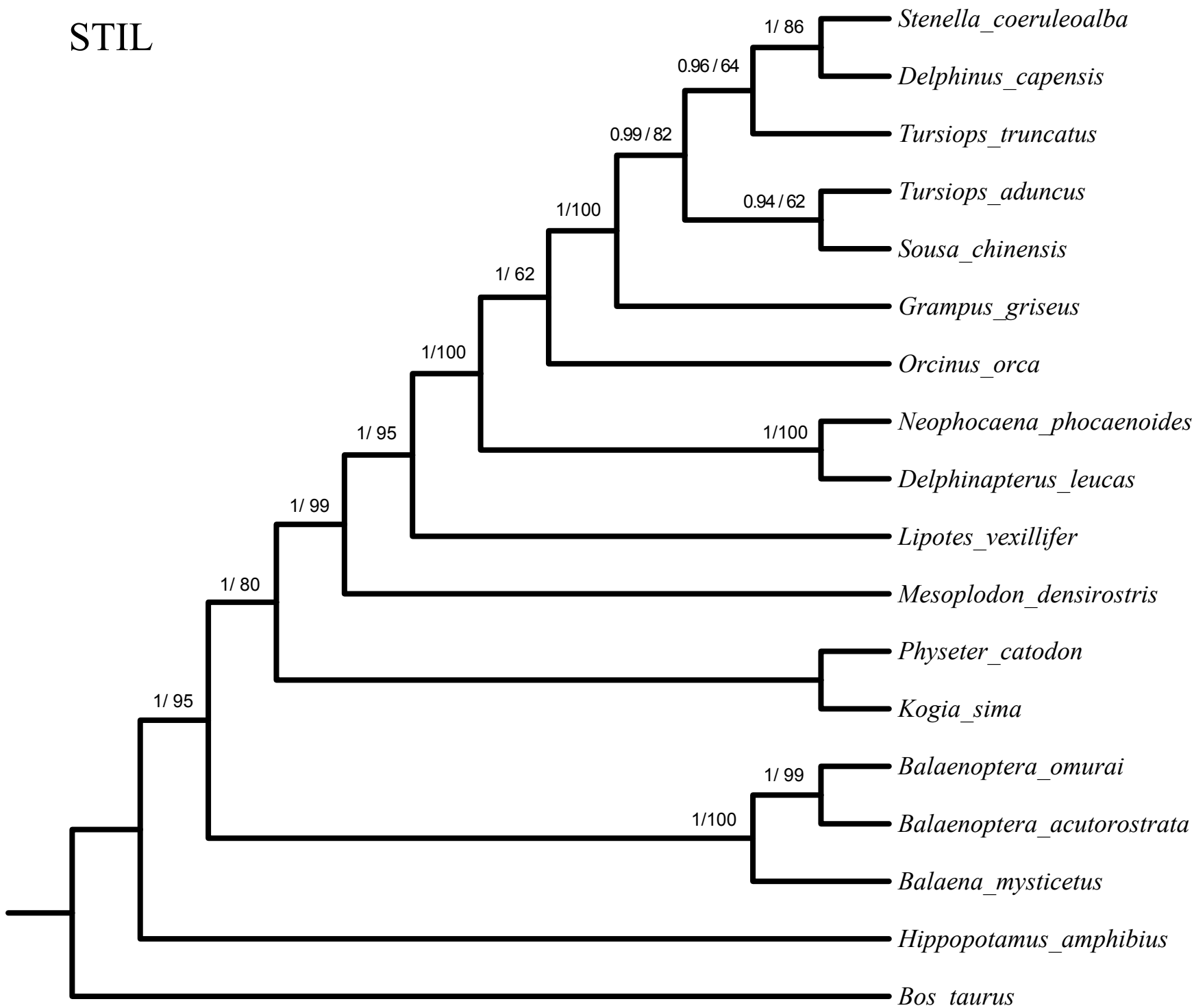

Supplement: Supplementary file 2 — Supplementary methods and results. Figure S1. Phylogeny of cetaceans based on ML and BI best topology; number above branches show bootstrap support and posterior probability value above 0.50. (ZIP 759 kb) [file 12862_2017_1051_MOESM2_ESM.zip › Fig. S1F Phylogeny of cetaceans at STIL.PDF]
