# Supplementary material for: Genetic basis of brain size evolution in cetaceans: insights from adaptive evolution of seven primary microcephaly (MCPH) genes
Source: BMC Evol Biol. 2017 Aug 29;17:206. doi: 10.1186/s12862-017-1051-7 (PMC5576371; doi:10.1186/s12862-017-1051-7)
Supplement: Supplementary file 3 — Alignment sequences of seven MCPH genes used for this study. (DOCX 60 kb) [file 12862_2017_1051_MOESM3_ESM.docx]

**Electronic Supplementary Information (ESM)**

**Genetic basis of brain size evolution in cetaceans: insights from adaptive evolution of seven primary microcephaly (*MCPH*) genes**

Shixia Xu*, Xiaohui Sun, Xu Niu, Zepeng Zhang, Ran Tian, Wenhua Ren, Kaiya Zhou, and Guang Yang*

Jiangsu Key Laboratory for Biodiversity and Biotechnology, College of Life Sciences, Nanjing Normal University, 1 Wenyuan Road, Nanjing, 210023, China

* Corresponding authors; e-mail: xushixia78@163.com and [gyang@njnu.edu.cn](mailto:gyang@njnu.edu.cn)

**Supplemental Methods and Results**

**Supplemental Methods**

**Association Analysis between Gene Evolution and Phenotypes**

Phylogenetic generalized least squares (PGLS) regression was used to analyze the relationship between log-transformed (root-to-tip ω) and each log-transformed morphological variable. We first reconstructed each node sequence of cetacean branches along Cetartiodactyla phylogeny implemented in the CODEML program of PAML 4.7 [23]. Second, we calculated the average “root-to-tip” ω along branches extending from the last common ancestor (LCA) of Cetacea to each extant cetacean species in the dataset. Root-to-tip ω was estimated by two-ratio branch models implemented in CODEML in the PAML 4.4 package [23]. Finally, we used PGLS regression to analyze the relationship between log-transformed (root-to-tip ω) and each log-transformed (morphological variable) or log-transformed (mean group size). The lambda (*λ*) value, estimated by maximum likelihood method [57], was used to quantitative measures of phylogenetic signal. The parameter *λ* can vary between 0 and 1: *λ* = 0 indicates there is no phylogenetic signal in the data whereas when *λ* = 1 suggest that the observed data strongly match expected phylogenetic patterns given a Brownian motion model of evolution. If there is no phylogenetic signal in the data, then PGLS will return estimates identical to an ordinary least square (OLS) analysis. If phylogenetic signal is intermediate, the model with better fit can be determined by the AIC value that the model with the lowest AIC value is considered the best fit of all regression models. All statistical analyses were performed in R 3.1.2 using the packages Caper [58].

**Functional Assays of the ASPM Gene**

Six species of odontocetes with increased EQ and three species of mysticetes with reduced EQ were chosen as representative samples. We cloned CaM into the pET-28a (+) expression vector to create an N-terminal hexahistidine-tagged fusion protein. The 23^rd^ IQ motif and its upstream 16 amino acid residues from nine cetacean species were amplified or synthesized (Sangon Biotech). We then cloned the motif into the expression vector pGEX-4T-1 to yield N-terminal tagged with glutathione-S-transferase (GST) fusion protein and verified it by sequencing (Sangon Biotech). Recombinant proteins were expressed in Escherichia coli strain BL21 (DE3) cells and purified by Ni2+- NTA or glutathione affinity chromatography, respectively, following the manufacture’s protocols. Purified proteins were further dialyzed against PBS overnight at 4 °C. For the GST pull-down assay, GST-IQ or GST proteins were mixed with glutathione-agarose and then incubated with purified CaM at 4 °C with gentle agitation for 2 h. Beads were centrifuged at 700 × g for 5 min and washed three times with PBS. The bound proteins were eluted by 10 mM reduced glutathione in 50 mM Tris-HCL, pH 8. The eluted samples were run on sodium dodecyl sulfate-polyacrylamide gels electrophoresis (SDS-PAGE) and subjected to western blotting by transferring the separated proteins onto a PVDF membrane and probing with a 1:1000 dilution of the anti-CaM antibody in 5% powdered milk / Tris-buffered saline, 0.1% Tween-20.

Binding affinities between CaM and GST-IQ were further quantitatively determined by biolayer interferometry (BLI) using the ForteBio Octet Red system. Briefly, biotinylated CaM at 5 nmol / L in PBS was loaded onto streptavidin (SA) biosensors for 10 min at room temperature. After reaching base line, SA biosensors were moved to the association step containing 62.5, 125, 250 and 500 n M GST-IQ fusion protein or GST control protein for 400 s and then dissociation for 500 s. All binding experiments were performed in duplicate.

**Phylogenetic tree reconstruct**

We reconstructed the phylogenetic tree of each intact MCPH gene using the maximum-likelihood (ML) and Bayesian inference (BI). The ML tree was constructed with the RAxML software (version 7.0.4) [59] using the standard default parameters with the GTR+G model of nucleotide evolution. The best ML topology and support values from 1000 rapid bootstrap replicates were calculated in RAxML using the CIPRES interface [59]. Bayesian inference methods with Markov chain Monte Carlo (MCMC) sampling were performed in MrBayes [60]. The analysis was run for 1, 000, 000 generations with a sample frequency of 1000 and burn-in was set to correspond to 25% of the sampled trees.

**Supplemental Results**

**Phylogenetic trees**

We reconstructed the gene tree for each MCPH gene using the maximum-likelihood (ML) and Bayesian inference. The result showed that the gene tree was completely identical to the well-accepted phylogeny of Cetartiodactyla [24] at the *CDK5RAP2* (Figure S1). By contrast, the phylogenetic relationships in the gene trees were similar to the well-accepted phylogeny with only some minor differences within Delphinidae at the *ASPM*, *WDR62*, *CEP152*, and *STIL* genes (Figure S1), including the position of the five dolphins, such as *Tursiops_truncatus*, *Tursiops aduncus*, *Sousa_chinensis*, *Grampus griseus*, and *Orcinus_orca*. Actually, the relationships within Delphinidae, especially within the Sousa-Delphinus-Tursiops-Stenella complex, remain controversial, despite that many studies have been conducted using a diverse array of systematic markers [61, 62, 63]. However, for CENPJ, the topological structure of the beaked whale (*Mesoplodon_densirostris*) was significant different to its “species tree” with this species at the base to all other cetacean lineages in the analysis (Figure S1).

For the selection analyses, we found that the selection detection using the gene trees produced results similar to those obtained using the well-accepted phylogeny of Cetartiodactyla (Table S6). This result was consistent with [25] suggested that the minor difference in the phylogenetic tree will not make any significant difference in estimation of parameters or identification of positively selected sites.

References

57. Pagel M. Inferring the historical patterns of biological evolution. Nature. 1999; 401:877-84.

58. Orme CDL, Freckleton RP, Thomas GH, Isaac NJB, Petzold T, Fritz SA. CAPER: comparative analyses of phylogenetics and evolution in R. Methods Ecol Evol. 2012;3:145-51.

59. Stamatakis A, Hoover P, Rougemont J. A rapid bootstrap algorithm for the RAxML web servers. Syst Biol. 2008,57:758-771.

60. Ronquist F, Huelsenbeck JP: MrBrayes 3: Bayesian phylogentetic inference under mixed models. Bioinformatics. 2003,19:1572-1574.

61. Xiong Y, Brandley MC, Xu S, Zhou K, Yang G. Seven new dolphin mitochondrial genomes and a time-calibrated phylogeny of whales. BMC Evol Biol. 2009;9:20.

62. McGowen MR, Spaulding M, Gatesy J. Divergence date estimation and a comprehensive molecular tree of extant cetaceans. Mol Phylogenet Evol. 2009, 53:891-906.

63. Caballero S, Jackson J, Mignucci-Giannoni AA, Barrios-Garrido H, Beltrán-Pedreros S, Montiel-Villalobos MG, Robertson KM, Baker CS. Molecular systematics of South American dolphins Sotalia: sister taxa determination and phylogenetic relationships, with insights into a multi-locus phylogeny of the Delphinidae. Mol Phylogenet Evol. 2008,46:252-68.
